# Sensory substitution of elbow proprioception to improve myoelectric control of upper limb prosthesis: experiment on healthy subjects and amputees

**DOI:** 10.1186/s12984-022-01038-y

**Published:** 2022-06-11

**Authors:** Matthieu Guémann, Christophe Halgand, Aurélia Bastier, Céline Lansade, Léo Borrini, Éric Lapeyre, Daniel Cattaert, Aymar de Rugy

**Affiliations:** 1 HYBRID Team, INCIA, CNRS, UMR 5287, Bordeaux, France; 2 Unité de Physiologie de l’Exercice et des Activités en Conditions Extrêmes,Département Environnements Opérationnels, Institut de Recherche Biomédicale des Armées, Brétigny, France; 3 Institut Robert Merle d’Aubigné, Valenton, France; 4 Physical and Rehabilitation Medicine Department, Percy Military Hospital, Clamart, France

**Keywords:** Amputation, Myoelectric, Proprioception, Sensory-substitution, Virtual reality

## Abstract

**Background:**

Current myoelectric prostheses lack proprioceptive information and rely on vision for their control. Sensory substitution is increasingly developed with non-invasive vibrotactile or electrotactile feedback, but most systems are designed for grasping or object discriminations, and few were tested for online control in amputees. The objective of this work was evaluate the effect of a novel vibrotactile feedback on the accuracy of myoelectric control of a virtual elbow by healthy subjects and participants with an upper-limb amputation at humeral level.

**Methods:**

Sixteen, healthy participants and 7 transhumeral amputees performed myoelectric control of a virtual arm under different feedback conditions: vision alone (VIS), vibration alone (VIB), vision plus vibration (VIS + VIB), or no feedback at all (NO). Reach accuracy was evaluated by angular errors during discrete as well as back and forth movements. Healthy participants’ workloads were assessed with the NASA-TLX questionnaire, and feedback conditions were ranked according to preference at the end of the experiment.

**Results:**

Reach errors were higher in NO than in VIB, indicating that our vibrotactile feedback improved performance as compared to no feedback. Conditions VIS and VIS+VIB display similar levels of performance and produced lower errors than in VIB. Vision remains therefore critical to maintain good performance, which is not ameliorated nor deteriorated by the addition of vibrotactile feedback. The workload associated with VIB was higher than for VIS and VIS+VIB, which did not differ from each other. 62.5% of healthy subjects preferred the VIS+VIB condition, and ranked VIS and VIB second and third, respectively.

**Conclusion:**

Our novel vibrotactile feedback improved myoelectric control of a virtual elbow as compared to no feedback. Although vision remained critical, the addition of vibrotactile feedback did not improve nor deteriorate the control and was preferred by participants. Longer training should improve performances with VIB alone and reduce the need of vision for close-loop prosthesis control.

## Introduction

The lack of feedback information, such as proprioception of a lost limb after an amputation, is devastating and has many consequences for patients. To operate a myoelectric prosthesis, a novel control system based on the activities of residual muscles has to be learned. Those prosthesis do not provide sensory information other than vision, onto which patients need to rely on to guide every movement [1, 2]. The lack of proprioceptive feedback has been identified as one of the main causes of prosthesis abandon, along with non-intuitive commands and insufficient functionality [3].

Since Childress (1980), it is known that the restitution of lost sensation is a key element in amputee rehabilitation [4]. Indeed, the literature and the patients report that vision provides a limited source of feedback [5, 6], especially in the context of object manipulation which is best relayed and integrated through tactile feedback [7]. To feed back a lost sensation, invasive and non-invasive methods have been explored. Invasive techniques include BCI using cortical electrodes that bypass the use of peripheral neural and musculoskeletal systems [6], and intraneural electrodes directly connected to a sensory nerve that could be stimulated to produce either tactile or proprioceptive sensation [8–11]. On the side of non-invasive techniques, one option is the integration of a sensory-substitution system, using another sensory modality to replace the missing one. This was initiated half a century ago and made famous by the work of Bach-y-Rita who recreated image by tactile stimulations on the skin of blind people [12]. This approach has been explored for patients with an amputation as a solution to restore a lost feedback which is considered as a priority as shown in [5]. The use of sensory feedback in a sensory substitution system has shown encouraging results for prosthesis control and acceptance when it conveyed information about contact, force level or object discrimination [13–18].

Proprioception (the sense of limb position and movement [19, 20]) is of primary importance in the execution of a motor task [21, 22]. However, this sense was much less studied than touch-based cutaneous feedback [16, 17, 23]. Few studies explored the use of either invasive or non-invasive approaches to restore proprioceptive feedback. One of the first work was realized by Mann et al. [24] who fed back the elbow joint position of the Boston arm through vibrotactile stimulation. They found that the display improved the subject’s accuracy and precision in positioning tasks. More recently, the sense of position and motion was fed back via a skin stretch device on healthy subjects [16], and results showed that average errors were lower with the device than with no feedback, but larger than with contralateral proprioceptive feedback. Moreover, participants had lower visual demand when using the device. Direct neural stimulation was also used to give feedback on limb position [9], and intraneural stimulation was also used to provide feedback for tactile and position information to improve accuracy control [11]. Results reported that neural stimulation allows the participant to estimate accurately join position, grip force, tactile cue and object shapes [9, 11]. In addition, sensory feedback has also been used to improve control of devices other than myoelectric prostheses. For example, Flesher et al. recently showed that tactile feedback restored with intracortical microstimulation of the somatosensory cortex reduced by two the trial times of the control of a robotic arm [6]. Taken together, these studies illustrate the growing interest of adding sensory signals to vision for motor control (for review see [23, 25, 26]).

These findings are encouraging, although most of the research involved healthy participants or was realized at the hand level to explore touch sense or grasp. Another limitation of these studies concerns the lack of a proper motor command associated with a task [16, 27]. The influence of being active and involved in a functional task is known to modify the sensory response to an action, and to improve perceptual performances when compared to a similar action delivered passively [28, 29]. Among studies performed on patients, only one involved an active myoelectric control task (box and block test) for participants [30]. This study of a functional grasping test showed that the addition of vibrotactile feedback improved the performance time and reduced the number of errors when visual feedback was disturbed [30].

The object of the present study was to explore the effect of giving a proprioceptive substitution signal (elbow positional angle information) on a functional task (moving the prosthetic elbow to a given angular position). To this aim we evaluated the influence of a novel vibrotactile feedback on the accuracy of myoelectric control of a virtual elbow by healthy subjects and participants with an upper limb amputation at humeral level.

Following recent work in which we showed that a circular arrangement of vibrors on the upper arm enables good spatial discrimination even with short (100ms) stimulus duration [31], we designed a sensory substitution system that fed back elbow proprioception with short alternating bursts of vibrors’ stimulations spatially arranged to match elbow angles. Participants performed discrete as well as back and forth elbow reaching movements under different feedback conditions, providing either vision alone (VIS), vibration alone (VIB), the addition of vision and vibration (VIS + VIB), or no feedback at all (NO).

## Methods

### Participants

Healthy participants were recruited from the research laboratory in Bordeaux (INCIA). Patients with a uni- or bilateral trans-humeral amputation were recruited from the Instruction Army Hospital of Percy and the Robert Merle d’Aubigné Institute of Valenton. Participants had to be over 18. Non-inclusion criteria were previous exposure to vibrotactile feedback, neurological of muscular affection, subject to epilepsy or any skin problem. Pregnant woman, people under 18, and person in custody or who cannot understand the protocol could not participate in the study. A medical check was conducted on the patients’ stump to detect any problem of superficial sensitivity (hyper-hypo sensitivity). All participants were informed about the content and goal of the study and signed a consent form. A national ethic committee approved the study which is registered with the number **IDRCB 2017-A03609-44**.

### Experimental set-up

**Fig. 1:**
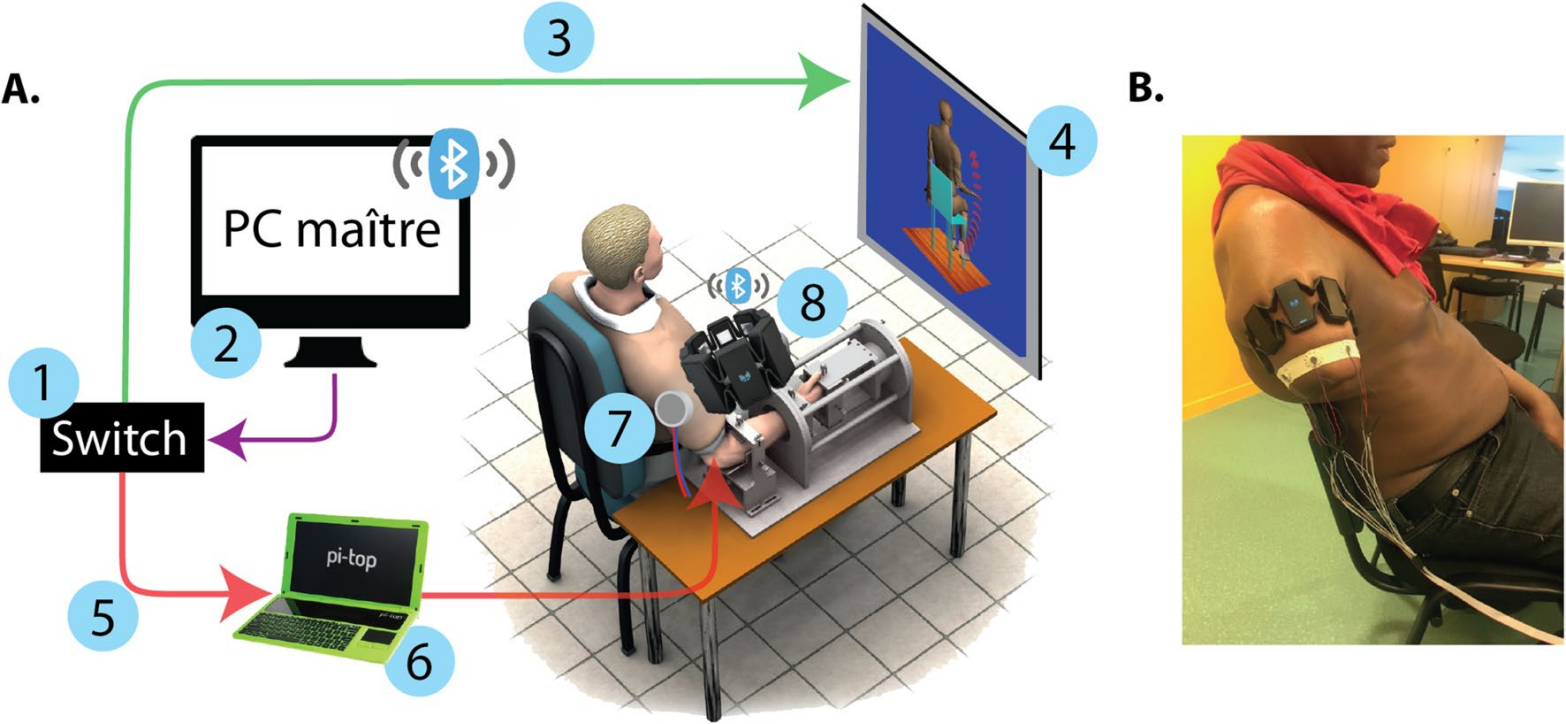
**A** experimental set-up with a healthy subject. (1) represents the switch allowing the communication between the 3 computers. (2) represents the command of the master PC sending the protocol instructions and evaluating in real time the avatar position. (3) represents the information transferred from the EMGs to move the avatar. (4) represents the computer where Animatlab was running, and the avatar displayed on a screen in front of the subject. (5) represents the commands sent from the master PC to the pi-top computer, receiving the exact arm position and converting it into an activation of the vibrors as the vibro-tactile feedback to the subject. (6) represents the pi-top computer where vibrors were connected. (7) represents the wristlet (also visible in Figure 1. **B** of 6 vibrors placed on the subject arm. (8) represents the MyoArm band used to collect the muscular activity (EMGs) and sent the data by a Bluetooth procedure to the master PC. B. subject with an amputation at the humeral level wearing equipment composed of the MyoArm band and the vibrors wristlet

The experimental set-up involved 3 interconnected computers, a wristlet containing the surface EMG (MyoArm®band) and another wristlet containing the vibrors (Fig. 1). Participants were seated in front of a TV screen showing a virtual avatar. The avatar’s arm movements were viewed from the side of the controlled arm (either left or right). Healthy subjects had their forearm fixed in order to emulate an isometric condition and avoid movement feedback from the elbow (Fig. 1A). Patients had their stump free (Fig. 1B). The recorded EMG activity of the biceps and triceps were used to control the flexion and extension of the virtual arm using a conventional velocity control mode. Muscle activities were collected at 200 Hz, rectified and filtered with a second order Butterworth filter with a cutoff frequency of 1.5 Hz.

Six vibrors of 7mm diameter and 2mm width (Seeed company) were used. They were placed circumferentially around the participant’s arm to elicit the best discriminable signal [31]. Each vibror was activated for a specific elbow angle from 20$^\circ$ to 120$^\circ$, with an inter-vibrors angle of 20$^\circ$ and a range of activation of 4$^\circ$ around each angle. The vibror’s sequence was 100ms activation interspersed by 100ms inactivation repeated as long as the participant stayed within the 4$^\circ$ range. When the participant stayed more than 2 consecutive seconds within the range, the inactivation period switched to 500ms to avoid discomfort. When the range of activation was left, the vibration stopped. The activation state of each vibror was updated as a function of the avatar’s arm position at a rate of 25 Hz (i.e., every 40ms).

### Preparation phase

#### Kinesthetic and visual imagery evaluation

Participants were evaluated for vividness of motor imagery with the Kinesthetic and Visual Imagery Questionnaire (KVIQ) adapted to our cases [32]. Although, the Initial questionnaire only evaluates the dominant upper limb, we repeated this section to test both upper limbs for healthy subjects, and both (amputated and non-amputated) upper limbs for patients. This represented a total of 26 questions instead of 13 with a maximum possible score of 130.

#### Vibror’s placement

Vibror’s wrislet was placed at the first third of the upper arm for healthy participant and at the stump level for the amputees. Vibror 1 was always placed medially to the biceps.

#### Spatial discrimination of vibrors

First, the vibrors were activated one by one to check for correct perception. The level of intensity of each vibror could be adjusted if the participant felt the stimulation as too strong or two weak. After this verification, a spatial discrimination task composed by two blocs of 24 random vibror activations (each vibror was activated 4 times) was conducted. The objective for the participant was to reach a 75% success rate score for one bloc to go further in the experiment. If success rate was lower than 75% by the end of the second bloc, the procedure was repeated once. Participants were seated and instructed to look in front of them. This way, they were not able to see the vibrors. The particular vibror felt by the participant as being activated was indicated verbally to the investigator who entered and validated the number associated to the vibror in a computer and launched the next stimulation. The advantage of this method, which we validated in a previous study [31], is that it places the emphasis on the vibror’s position rather than on the mere distinction between two vibrors as in a more classical force choice task [33–35].

#### Myoelectric control calibration

Myoelectric signals were recorded from the Myo armband which is recognized for its ease of use and precision despite its low cost [36] Participants were asked to contract alternatively their biceps and triceps. Two amongst 8 electrodes which enable the best muscle dissociation were selected. Then, participants produced 2 seconds maximal voluntary contraction (MVC) from each muscle (biceps and triceps). These MVC were used for EMG normalization. The normalized signals were used to control the elbow rotation velocity of the virtual arm according to a conventional velocity-based myoelectric control [37, 38]. Participants were able to test it for speed and precision during few minutes and eventually adjusted MVC threshold and velocity gain.

### Experimental phase

This phase was composed of two parts. In the first part, participants had to realize discrete movements of different magnitudes between two targets with the virtual arm. In the second part, they had to realize back and forth movements of different magnitudes between two targets, with a clear stop between each change of direction. In both parts, the time allowed to execute the movement was proportional to the distance between the targets. At the end of each part, a test was conducted to compare the accuracy of the movement in 4 different feedback conditions which were vision alone (VIS), vision and vibration (VIS + VIB), vibration alone (VIB) and no feedback at all (NO).

#### First part, discrete movements

This part was composed of 2 exercises and 1 test. In the first exercise, participants produced 30 movements (flexion or extension). The first set of 10 movements was produced with the visual feedback only, the second set with the visual and vibrotactile feedbacks, and the third set with the vibrotactile feedback only. Movements’ initial and final target positions were randomly selected from a list within the range of 20$^\circ$ to 120$^\circ$, and a step of 20$^\circ$. After each movement, the distance to the target during the last 500ms was feedback. The appreciation “good” was given for movement within a range of ±4$^\circ$ to the target, “almost” if between 4$^\circ$ and 10$^\circ$, or “wrong” if over 10$^\circ$. At the end of each series, the results over the last 10 trials were shown to participants. The second exercise was designed to help participants to associate their muscular activity with the different positions corresponding to each vibror activation. In a random order, participants were asked to activate each of the 6 vibrors which correspond to 6 different targets. This was repeated 4 times per vibror (24 movements in total). For each trial, they had 12 seconds to reach the correct vibror. The starting position was always the position 0$^\circ$, in which the avatar’s arm was fully extended. During all trials, the avatar was hidden (black screen) and participants only see the number of the vibror they had to activate. At the end of each trial, the trajectory of the realized movement was shown. After those two exercises, the participants performed a test session composed of 35 movements organized in 10 blocks of 3 movements (one per type of feedback) and 5 movements without any feedback at all (i.e., black screen with no vibration). The movement without feedback occurred every two blocks. At the end of each trial, the result was given based on the same method as in the first exercise (good, almost, wrong).

#### Second part, back and forth movements

In the second part, participants realized back and forth movements between two targets with a clear stabilization (movement of less than 2$^\circ$ during 500ms) at each change of direction. As in the first part, participants started with an exercise composed of 3 sets of 10 trials where each set was composed of movements with one type of feedback (visual, visual and vibrotactile, and vibrotactile only). At the end of each trial, the trajectory of the realized movement was shown. After this exercise, participants performed a test composed of 8 blocks of 3 movements (one per type of feedback) presented in a random order, interspersed with 4 trials (one every two blocs) without any feedback at all (i.e., black screen with no vibration).

#### Workload evaluation with NASA-TLX and preference ranking

At the end of the experiment, healthy participants were asked to fill a NASA-TLX auto-questionnaire for each type of feedback experienced (visual, visual + vibrotactile, and vibrotactile only) [39–41]. This evaluates factors influencing the workload. As a final step, healthy participants were also asked to rank the 3 types of feedback according to their preference. Please note that due the small number of patients involved and experimental constraints, these analyses were not conducted on patients.

### Statistical analysis

#### KVIQ test

Between groups comparison of the scores for amputee and non-amputee participants was made using the non-parametric Mann and Whitney test.

#### Spatial discrimination test

A minimum of 2 discrimination tests were realized before the experimental phase. The rate of correct answer between the 2 tests was analyzed with the Wilcoxon signed-rank test to see if an evolution occurred between the first and the second test or the last two tests across participants.

#### Effect of the feedback type on movement accuracy

For discrete movements, statistical analyses were performed on the absolute differences between the final elbow angle and the target angle, averaged for each subject and each feedback condition. For the back-and-forth movements, the absolute differences between elbow angles and target angles were computed on the first 6 stabilization periods (i.e., changes of movement direction during the first 3 back and forth movements) and averaged for each participant and feedback conditions. As the data did not pass the test for normality, a non-parametric Friedman test was first used to detect a main effect of feedback type on movement accuracy. When a main effect was found, a post-hoc analysis using Wilcoxon signed-rank tests with a Bonferroni correction was applied for two-by-two comparisons between specific feedback types.

#### NASA-TLX

A Friedman test was conducted on scores obtained at the NASA-TLX questionnaire, followed by two-by-two comparisons between feedback types conducted using Wilcoxon signed-rank tests with a Bonferroni correction.

All statistical tests were conducted using Python 3.6 with scipy.stats and pingouin modules [42], with a threshold for statistical significance set at $\alpha$ = 0.05.

## Results

### Participants

Sixteen healthy subjects (including 6 women) with a median age of 26 and 6 patients (all men and one with a bilateral amputation) with a median age of 60 participated to the experiment. Period since the amputation varied from 1 to more than 40 years (Tables 1 and 2). Most patients wore a myoelectric prosthesis for the hand, whereas the prosthesis’ elbows were either mechanic or myoelectric. One patient did not use his prosthesis anymore due to a pain episode at the stump. One patient was bi-amputated and realized the experiment twice with his right and left stump.

**Table 1:** Anthropomorphic data of healthy participants

Subject	Sex	Laterality	Age (years)	Arm circumference (cm)
1	Male	Right	29	32
2	Female	Right	25	25
3	Female	Right	26	27
4	Male	Right	26	30
5	Male	Left	30	25
6	Female	Right	26	27
7	Male	Left	27	31.5
8	Female	Right	25	25
9	Male	Right	28	27
10	Female	Right	26	25
11	Male	Left	29	27
12	Male	Right	27	30
13	Male	Right	22	28
14	Male	Right	23	28.5
15	Female	Left	27	23.5
16	Male	Left	23	26

**Table 2:** Anthropomorphic data of patients

Subject	Sex	Laterality before amputation	Laterality after amputation	Side of amputation	Age (years)	Stump circumference (cm)	Stump circumference (cm)	Type of prosthesis	Time since amputation
1	Male	Left	Left	Right	57	24	21	Myoelectric	41
2	Male	Right	Right	Left	60	23	25	No (pain)	1
3	Male	Right	Right	Left	35	19	26	Myoelectric	9
4	Male	Right	Right	Left	48	30	20	Aesthetic 3	
5	Male	Right	Right	Right-Left	24.5	17	Myoelectric	2	
6	Male	Right	Right	Right-Left	27	21	Myoelectric	2	
7	Male	Left	Left	Right	65	25	22	Myoelectric	21

### KVIQ test

Mean scores were 105.44 (SD= 15.35) and 97.57 (SD= 16.50) for healthy subjects and patients, respectively. A score over 78 (mean of 3 for each item) is considered good [32]. No statistical difference was found between groups (Q= 71.5, p= 0.315).

### Discrimination test

**Fig. 2:**
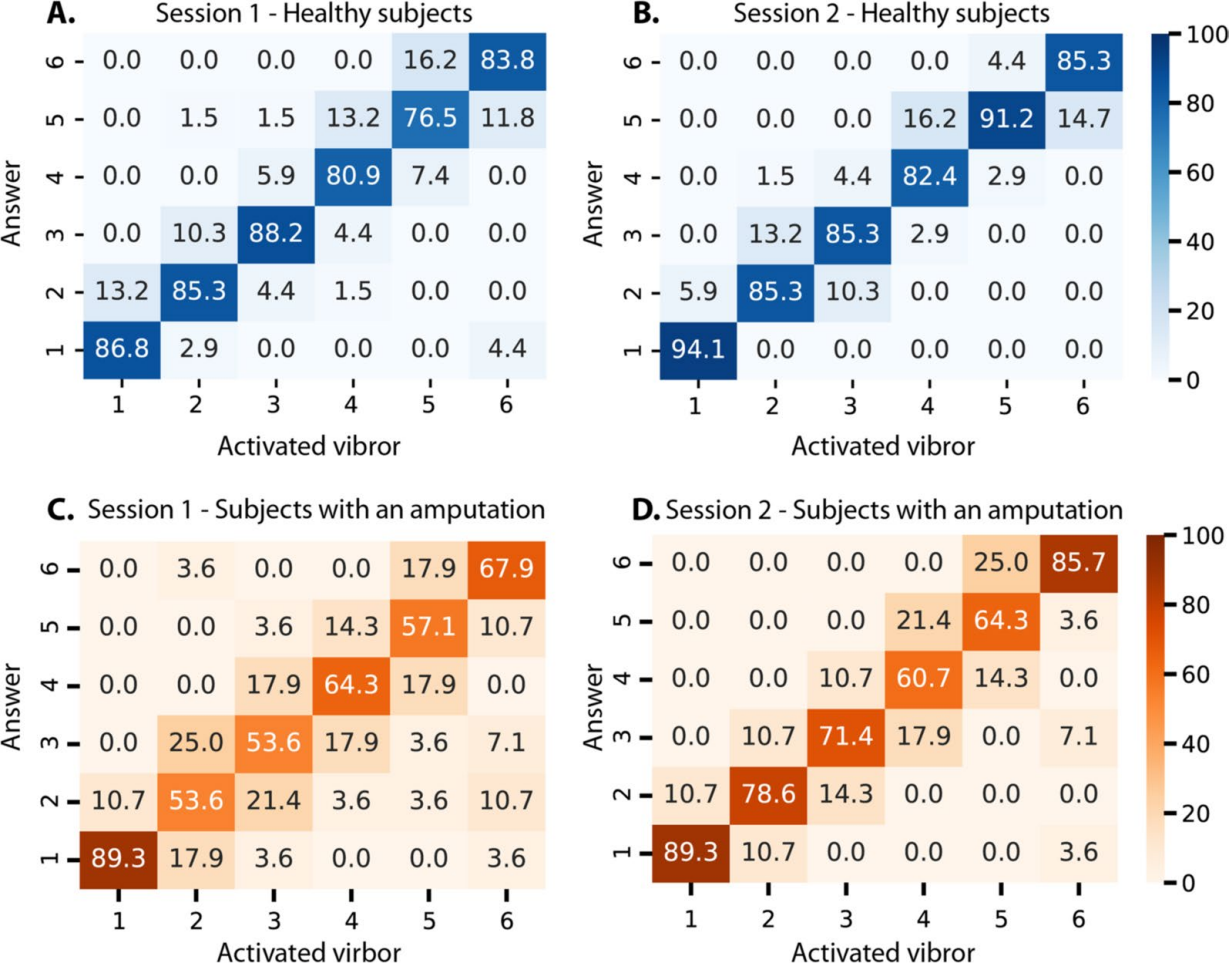
**A** and **B** confusion matrices for healthy subjects showing the rates of correct answers for the first and the second discrimination test for healthy participants. Correct answers are represented on the diagonal where the activated vibror (x-axis) is the same as the answer vibror (y-axis). **C**, **D** show the confusion matrices answer rates of the discrimination test for subjects with an upper arm amputation

Confusion matrices of the response rate per vibror of the first and second discrimination tests for healthy participants and patients are shown in Fig. 2. Healthy participants could recognize each vibror with a success rate above 75% for the first test, and above 80% for the second. When participants made mistakes, it was mostly toward an adjacent vibror. No statistical difference was found between the first and second tests (W = 31.5, p = 0.35). For patients, the average scores were significantly lower on the first test (67.72%) than on the second test (83.33%) (W = 0.0, p=0.02).

### Movement Accuracy

**Fig. 3:**
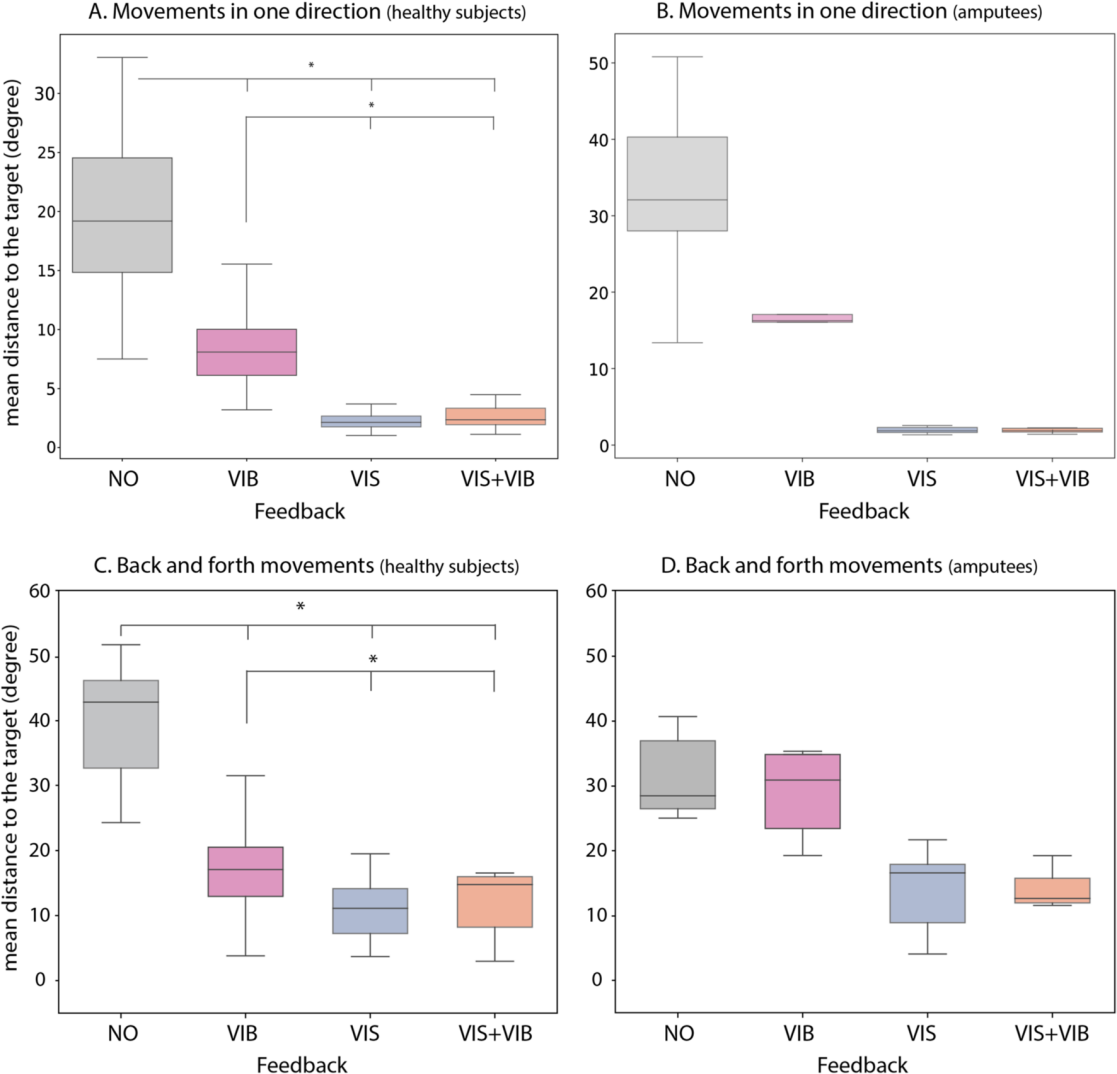
**A**, **B** Boxplot showing the dispersion by quartile of the mean of the absolute difference from the avatar’s hand to the target after movements in one direction for healthy subjects and subjects with an amputation. Feedback condition are no feedback at all (NO), vibration only (VIB), vision only (VIS) and the combination of vision and vibration (VIS+VIB) for healthy **A** and amputees **B**. **C**, **D** shows the boxplot of the dispersion by quartile of the mean of the absolute difference from the avatar’s hand to the target for maximum and minimum scores during back-and-forth movements for healthy **C** and amputees **D**

The Friedman test revealed an effect of the feedback type on the accuracy (i.e., absolute angular error) of discrete movements both for healthy participants and patients (Q = 43.5 and 14.04, respectively, p<0.05) (*See supplementary data*). For healthy subjects, two by two comparisons showed statistical differences between the NO condition and the three others, and between the VIB condition and the 3 others (Fig. 3A). No difference was found in any of the two-by-two comparisons conducted on patients (Fig. 3B). The patient with a bilateral amputation only realized this exercise with the right arm (arm of laterality).

For the back-and-forth movements, the Friedman analysis also revealed an effect of the feedback type for both healthy participants and amputee (Q=29.16 and 12.12, respectively, p<0.05). Two by two comparisons on healthy participants’ data revealed statistical differences between the condition NO and the three other conditions, and between the conditions VIB and VIS (Fig. 3C). Still for healthy participants, no difference was found between conditions VIB and VIS &VIB, nor between conditions VIS &VIB and VIS. For the two-by-two comparisons conducted with patients, no difference was found between any feedback type (Fig. 3D). Note that only 5 patients realized this part of the experiment (IB09 didn’t due to fatigue). Altogether, Fig. 3 indicates a global pattern with higher errors in the NO feedback condition than in the VIB condition, showing that our vibrotactile feedback improved performances as compared to no feedback at all. Yet, condition VIB typically elicits higher errors than conditions VIS and VIS &VIB, the latter two eliciting similar performances. Vision appears therefore critical to maintain a good level of performance, which is not ameliorated nor deteriorated by the addition of vibrotactile feedback.

### Workload and preference

**Fig. 4:**
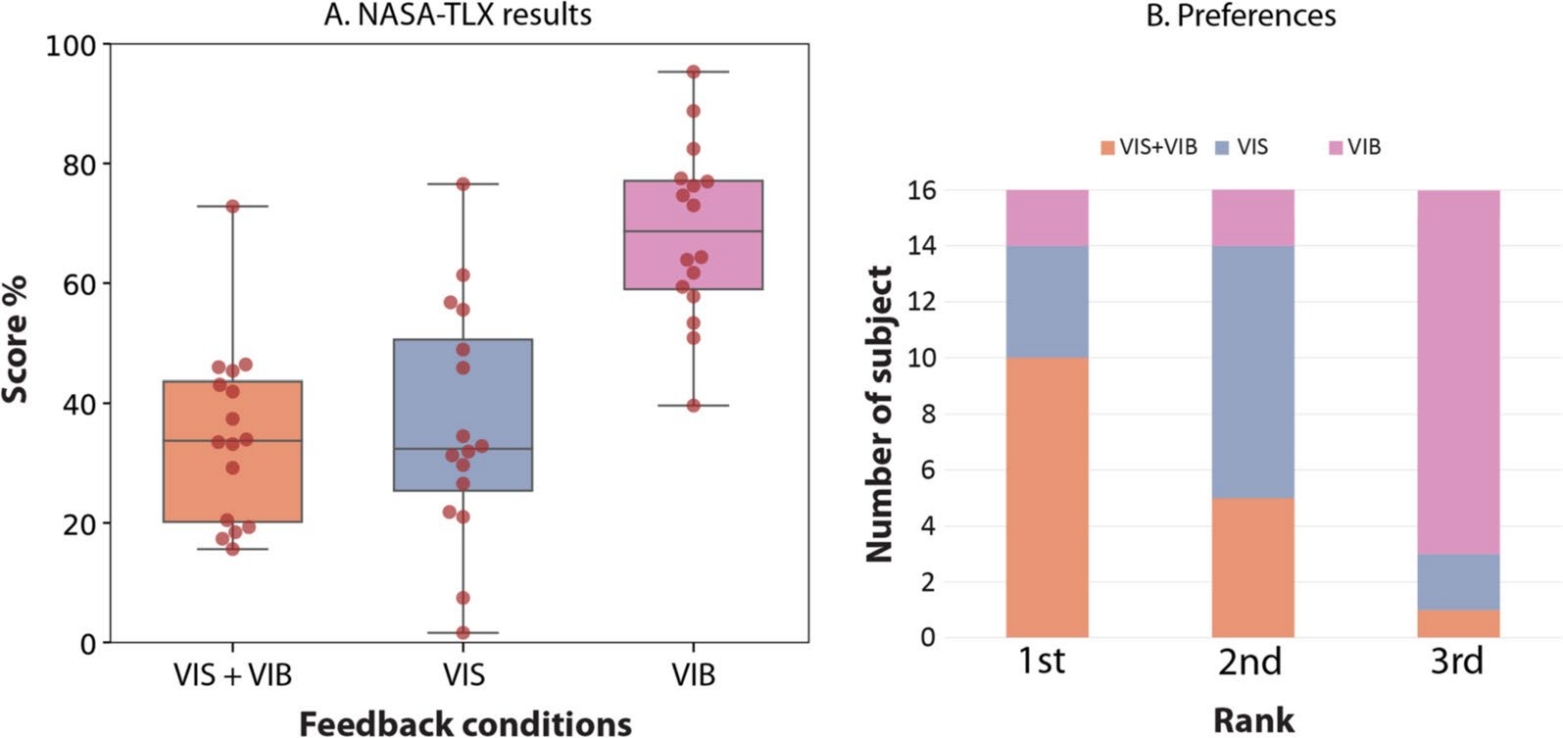
**A** Boxplot showing the dispersion by quartile of the results obtain at the NASA-TLX questionnaire filled by healthy subjects for the different feedback conditions. Each dot represents a participant. **B** Raking of the different feedback conditions from the preferred (1st) to the worst (3rd) by healthy participants

Mean (SD) scores at the NASA-TLX questionnaire conducted on healthy participants were 39.03 (19.58) for VIS, 37.20 (14.17) for VIS+VIB and 70.83 (14.25) for VIB (Fig. 4A). The Friedman test revealed a main effect of feedback conditions (Q=19.00; p<0.0001), and two by two comparisons revealed significant differences between VIS and VIB (W=1.0; p<0.001) and between VIB and VIS &VIB (W=0.0; p<0.001), but no difference between VIS and VIS &VIB (W=53; p=0.47).

Additionally, Fig. 4B indicates how healthy participants ranked the 3 feedback conditions according to their preference. This revealed that a majority of participants ranked the VIS &VIB condition first (10/16), the VIS condition second (9/16), and the VIB condition last (13/16). Vibrotactile feedback added to vision was therefore preferred, although vision only was preferred to vibrotactile only. As indicated in the method, these analyses were not conducted on amputee participants.

## Discussion

Here, we designed and evaluated a novel vibrotactile feedback encoding elbow proprioception to evaluate the effect on myoelectric control of a virtual arm by healthy participants and amputees. Results show that the vibrotactile feedback improved myoelectric control as compared to a condition without any feedback at all and did not improve nor deteriorate the control accuracy when added to vision as compared to vision alone. Furthermore, if the vibrotactile feedback alone increased workload as compared to vision alone, it did not so when added to vision. Finally, the vision plus vibro-tactile feedback was preferred my most participants.

Referring to Bayesian principles of multisensory integration, the use of multiple sources of information weighted according to their degree of certainty should improve global performance as compared to that with single sources of information [43–46]. Thus, we expected vibrotactile feedback added to vision to improve performance as compared to vision alone. This was not the case, as performances obtained in both conditions did not differ. Those results could be explained by different factors, such as the complexity of the task and the associated precision of a putative feedforward internal model [47]. As our experiment focused only on one degree of freedom, our sensory substitution system might not have given enough additional information to vision in order to significantly improve the myoelectic control. Indeed, previous work demonstrated that position estimation based on vision is very precise, whereas speed estimation based on vision has a much higher discrimination threshold [48]. In this context of poor speed estimation by the visual system, Earley et al. showed that sensory substitution based on auditory feedback was able to decrease speed uncertainty when paired with vision. This led them to propose that *“if artificial feedback can’t match visual precision, it will be largely ignored in favor of vision”* [48]. In fact, this might very well be what has been happening in our VIS+VIB condition, as well as in Pisthol and al. who also evaluated the addition of artificial proprioception to vision in order to improve myoelectric control [27]. In this study, participants experienced 4 types of feedback which were vision alone, vision and artificial proprioception, artificial proprioception alone and no feedback. Artificial proprioception was given by a robotic manipulandum which guided the participants’ hand along the trajectory controlled by the myoelectric activity of their other hand. In one experiment, the authors found that *“no improvement over visual feedback could be found in the visual + proprioception feedback condition (...) but that proprioception feedback alone was consistently better than without feedback”*. These findings attest the potential and utility of proprioception feedback, but also show how strong the visual information is. Our findings, similar to these of Pisthol et al. [27], confirm what Mon-Williams et al. [49] previously commented: *“we believe in what we see, rather than in what we feel, when the visual background is rich, and in what we feel when the visual background is sparse.”* Future experiments might explore conditions that could promote the implication of proprioceptive feedback, such as reaching objects that are out of sight or only via peripheral vision (e.g., while fixating to an opposite virtual target), and/or dual tasks where the cognitive workload is increased [50].

In addition to motor performance, our study points out the preference for the combined feedback condition (VIS + VIB) (as was also observed by Pistol et al. [27]) for most healthy participants (10 out of 16), despite their first and unique exposure to this condition. This self-reported preference for the combined (multisensory) condition shows the potential usefulness of the integration of a sensory-substitution system in the motor command of myoelectric devices. Moreover, participants reported that vibrotactile feedback was useful to confirm the avatar’ positions reached under myoelectric control. Yet, our experimental set-up and design might not have been sensitive enough to detect behavioral changes that would be associated with such an increased role of proprioception. Indeed, finding outcome metrics that are sensitive to capture the functional impact of sensory feedback and/or proprioceptive systems remains challenging. This is highlighted by recent DARPA investment in peripheral nerve interfaces, where an entire section is dedicated to the evaluation and efficacy of the system [51]. Aside of functional metrics and user feedback, new approaches combining mathematics, psychophysics and theory led to develop novel metrics that should be useful for future research to quantify the benefit of the new sensation [52].

In the present report, we observed performance improvements, although participants were tested in a single session of practice. These results, although limited, are encouraging if we consider that they were obtained without training. Indeed, several sensory substitution studies relied on multiple sessions to elicit performance improvements [53–55], which indicates that our observations from a single session should improve further following training. The workload associated with the vibrotactile feedback points toward this interpretation. The higher workload associated with vibrotactile feedback alone as compared to vision alone suggests that an additional cost was associated with the integration of the novel feedback. This reminds us of a typical learning process where efforts are needed at the beginning to integrate new rules and functions. Although this additional cost could have deteriorated the performance of the vision plus vibrotactile feedback condition, it is encouraging that this was not the case, and that the combined feedback condition did not increase the control workload neither. The absence of overload for participants using the sensory substitution system presented here are therefore encouraging for future research that should include further training.

The good performance maintained with the addition of the vibrotactile feedback and the preference for the multimodal condition could be explained by the congruency between the feedback signal and the information it delivers. This congruency has been reported as a key element for the use and integration of a sensory-substitution system [56]. In fact, it has been shown that when the feedback signal is not congruent or is in conflict with vision, it is not integrated in the motor control strategy [27, 57]. Here, although our feedback was not modality-matched in the sense of a stimulus that would be felt in the same modality as the initial sensory information [25, 58], it was designed to be as intuitive as possible, with elbow rotation directly translated into a rotation of the vibration around the arm. In Guemann et al. [31], we showed that tactile perception was better with this circular arrangement of vibrors on the arm than with a longitudinal one, probably due to the increased likelihood of stimulating different dermatomes and mechanoreceptive units whose oval-shaped receptive fields are oriented in the longitudinal axis [59, 60]. As tactile perception was also found efficient with short vibrotactile stimulations in that study (100ms), successive discrete bursts of vibration were designed here to transmit proprioceptive information relevant to closed-loop motor control. While discrete tactile feedback has already shown clear benefits in the context of prosthesis control [53, 61], we believe that our sequence of discrete bursts that vary both in space and time according to arm movements offers additional perspectives for future research.

Our experiment shows a novel type of non-invasive sensory substitution feedback system that could be easily implemented and used with upper-limb amputees. In addition to the advantages reported for non-invasive feedback modalities [53, 62], the vibrors wristlet used here was easily adaptable for each participant with a variable arm circumference, and the small space occupied by the vibrors is such that they could easily be integrated into a prosthetic socket. Although vibrotactile stimulations get growing attention, electrotactile stimulations or even hybrid (vibrotactile and electrotactile) stimulations are also used for tactile, force or proprioceptive feedback [54, 63, 64]. With respect to vibrotactile feedback, the ease of use, the small size and the small power consumption are presented as its best advantages [26]. However, some limitations related to unpleasant feeling and interference with EMG sensors are also highlighted and should be taken into consideration [26, 64]. Promising alternatives include invasive techniques such as direct nerve stimulations, which could elicit tactile sensation [1, 10], and proprioception [8]. However, the actual use of such devices is somehow limited by the surgical procedure involved, the potential nerve damage and the limited lifetime of the implant [26].

Together with the advantages associated with the use of a non-invasive device, our approach provides perspectives for further improvements. A first one is related to practice and familiarization period in relation to the vibrotactile signal. Participants had a single session with only few minutes to identify, understand and map the signal. As a comparison, in the experiment of Strbac, participants had to learn to discriminate vibro-tactile feedback that corresponded to 4 levels of grip strength [65]. The training protocol included 5 training sessions spread over 5 consecutive days where each session consisted of 4 blocks of 60 trails. As a result, each participant had produced about 1200 trials over a week of training, where, in our experiment, only 147 trials were realized. Our results are therefore encouraging as they reveal that our protocol of alternating short bursts of vibrors’ stimulations is somewhat immediately understood and useful to participants, such that longer rehabilitation periods are very likely to elicit further improvements.

Regarding the task, the fact that regions translated by vibrors were aligned with target positions might also impact the generalizability of our results. Indeed, some participants could have counted the number of activated vibrors required to achieve the target rather than identifying it more directly from the pattern of vibrations. In practice, this strategy would be difficult to apply due to jumps of activated vibrors generated by strong muscle contraction. It remains that our success rates could have been influenced by participants using this strategy while slowly executing their movement. To assess this “counting” effect in future work, one might manipulate systematically the offset between target and feedback positions.

In our study, a relatively small number of amputees were included (six upper limb amputees, one of them being bi-amputated), that were heterogeneous in term of age, prosthesis habits and usage. Surprisingly, neither laterality, age nor stump length and circumference seemed to have limited the use of our device. The wristlets fitted all participants, and no discomfort has been reported. One of the main advantages of this type of device is that it could be used early on in the rehabilitation period, possibly at home even before the first prosthesis is actually fitted to the patient. In addition, finding relevant candidates is a common difficulty encountered in other publications. In their works, Markovic, Witteveen and Strbac only included 5, 7 and 9 trans-radial participants, respectively, to test a vibrotactile device [13, 47, 65]. Our objective was even more difficult as we included trans-humeral amputees. As an interesting perspective, adapting our interface to trans-radial amputees could enable increasing the number of participants and explore the benefit of our novel feedback for different gestures such as opening/closing a virtual hand or control grip forces.

## Conclusion

Here we propose a new simple and comprehensive way to feedback proprioception using intact sensory pathway available on the patient’s skin. Previous studies revealed that the sooner and the longer the training period, the better the effect on cortical representation while avoiding maladaptive plasticity [66, 67]. Myoelectric training combined with vibrotactile stimulation might also have positive effects on phantom limb pain, which concerns most patients and currently suffers from a lack of efficient treatments [68, 69]. This motivates further explorations of online myoelectric control with vibrotactile feedback such as the one proposed here.

## Data Availability

The datasets used and/or analysed during the current study are available from the corresponding author on reasonable request.
